# Quantum-Inspired Moth-Flame Optimizer With Enhanced Local Search Strategy for Cluster Analysis

**DOI:** 10.3389/fbioe.2022.908356

**Published:** 2022-08-10

**Authors:** Xinrong Cui, Qifang Luo, Yongquan Zhou, Wu Deng, Shihong Yin

**Affiliations:** ^1^ College of Artificial Intelligence, Guangxi University for Nationalities, Nanning, China; ^2^ Guangxi Key Laboratories of Hybrid Computation and IC Design Analysis, Nanning, China; ^3^ Xiangsihu College of Gunagxi University for Nationalities, Nanning, China; ^4^ College of Electronic Information and Automation, Civil Aviation University of China, Tianjin, China

**Keywords:** K-means, cluster analysis, quantum-inspired moth-flame optimizer, local search mechanism, swarm intelligence

## Abstract

Clustering is an unsupervised learning technique widely used in the field of data mining and analysis. Clustering encompasses many specific methods, among which the K-means algorithm maintains the predominance of popularity with respect to its simplicity and efficiency. However, its efficiency is significantly influenced by the initial solution and it is susceptible to being stuck in a local optimum. To eliminate these deficiencies of K-means, this paper proposes a quantum-inspired moth-flame optimizer with an enhanced local search strategy (QLSMFO). Firstly, quantum double-chain encoding and quantum revolving gates are introduced in the initial phase of the algorithm, which can enrich the population diversity and efficiently improve the exploration ability. Second, an improved local search strategy on the basis of the Shuffled Frog Leaping Algorithm (SFLA) is implemented to boost the exploitation capability of the standard MFO. Finally, the poor solutions are updated using Levy flight to obtain a faster convergence rate. Ten well-known UCI benchmark test datasets dedicated to clustering are selected for testing the efficiency of QLSMFO algorithms and compared with the K-means and ten currently popular swarm intelligence algorithms. Meanwhile, the Wilcoxon rank-sum test and Friedman test are utilized to evaluate the effect of QLSMFO. The simulation experimental results demonstrate that QLSMFO significantly outperforms other algorithms with respect to precision, convergence speed, and stability.

## 1 Introduction

Clustering is the process of grouping objects into clusters according to the similarities within the data objects (Jain et al., 1999). Cluster analysis (Jain, 2010) does not need to refer to any classification information beforehand and can classify data by judging the similarity of data features. So, clustering belongs to unsupervised learning in machine learning (Sinaga and Yang, 2020). It is widely used in customer classification (Deng and Gao, 2020; Li et al., 2021a; Sun et al., 2021), automatic medical image detection (Hassan et al., 2021), image retrieval (Karthikeyan and Aruna, 2013; Gu et al., 2019; Anju and Shreelekshmi, 2022), object recognition (Woźniak and Połap, 2018), data mining (Hosseini et al., 2010), (Sato et al., 2019), pattern recognition (Xu et al., 2022), (Singh and Ganie, 2021), and other fields.

There are four taxonomic methods applied to cluster analysis: partition-based, hierarchical, grid-based, and density-based methods. K-means is a partition-based method that is widely welcomed owing to its simple calculation steps and easy implementation. However, the clustering effect obtained by the K-means is easily influenced by the preliminary location of the centroids and is vulnerable to the risk of slipping into a local optimum as the algorithm proceeds. There are two solutions to optimization problems, one is deterministic and the other is non-deterministic. Real-life engineering optimization problems have the characteristics of complexity, large-scale, nonlinearity, multiple constraints, and high dimensionality. However, deterministic methods can often not effectively calculate the correct results for such optimization problems. Therefore, it is an urgent matter to find efficient ways to solve optimization problems. Researchers have used non-deterministic algorithms, also known as stochastic optimization algorithms, to achieve excellent results in solving certain real-world complex optimization problems over the last decades. Meta-heuristic intelligent optimization algorithms fall under the category of stochastic algorithms, and researchers have successively proposed many intelligent optimization algorithms, which have become a very popular and feasible solution in dealing with complex problems. Classical metaheuristic algorithms that have been widely used include differential evolution (DE) (Storn and Price, 1997), grey wolf optimizer (GWO) (Mirjalili et al., 2014), particle swarm optimization (PSO) (Kennedy and Eberhart, 1995) and Monarch butterfly optimization (MBO) (Wang et al., 2019), etc. Many researchers have made a variety of meaningful improvements to promote the performance of the algorithm, such as Wei et al. (2021) proposed an improved slime mold algorithm with oscillating factor and Levy flight strategy for optimal reactive power dispatch problem. Wang and Tan (2017) proposed a method based on the information feedback model to improve the performance of the heuristic algorithm. Wang et al. (2014) introduced chaos theory into the optimization process of the krill herd (HK) algorithm, and the algorithm’s performance was effectively improved. Gao et al. (2020) used an improved DE algorithm with a selection mechanism to solve the fuzzy job-shop scheduling problem. Merwe and Engelbrecht successfully combined the PSO to address the clustering problem by constructing the structure of the evaluation function and the solution (Van der Merwe and Engelbrecht, 2003). Wang et al. (2016) improved the precision and convergence rate of the flower pollination algorithm on cluster analysis by adding the discard pollen operator. Zhou et al. (2017) propose an improved social spider optimization algorithm that introduces a stochastic strategy known as the simplex method to deal with clustering analysis. A symbiotic biological search algorithm for clustering analysis has been proposed by Zhou et al. (2019). Ouadfel and Abd Elaziz (2021) introduced an improved multi-objective gradient optimizer to handle the clustering problem of multi-view datasets. Taib and Bahreininejad (2021) introduced an improved water cycle algorithm incorporating an algorithm for evaporation rate to tackle the clustering analysis problem. Wang et al. (2021) implemented a meme algorithm with adaptive inverse K-means operation to tackle the clustering question.

The Moth-flame Optimizer (MFO) (Mirjalili, 2015a) is a firmly established meta-heuristic optimization algorithm that has proven to be efficient and potentially capable of addressing real-life problems. After MFO was put forward, many scholars applied it to various engineering problems and achieved good results. Elsakaan et al. (2018) applied a moth flame optimizer to solving economic scheduling problems. Elaziz et al. (2020) introduced an OMFODE algorithm that integrates opposition learning strategy and differential evolution algorithm for the feature selection problem. Moreover, the classification of galaxy images is successfully implemented with satisfactory results. Khan et al. (2021) used MFO to optimize the integrated power plant system containing stochastic wind. Ahmed et al. (2021) applied MFO to optimizing workflow scheduling in fog computing. Li et al. (2021b) used opposition-based learning (OBL) and differential evolution (DE) algorithm to improve the quality of the flame population to enhance the efficiency of the standard MFO. Wu et al. (2022) construct a bi-clustering-based moth-flame optimizer for recommender systems to successfully generate recommendation lists and predict unrated items for target users.

Quantum computing (QC) integrates concepts from three disciplines: quantum physics, computer science, and classical information theory (Steane, 1998). At present, more and more researchers combine quantum computing with heuristic algorithms and try to apply them in various fields. Han and Kim (2002) were the earliest to combine QC with evolutionary algorithms to solve combinatorial optimization problems. Layeb (2011) applied a cuckoo search algorithm combined with quantum-inspired for knapsack issues. Cai et al. introduced the simulated annealing (SA) strategy and the quantum revolving gate (QRG) strategy into the moth flame optimizer to improve the local development and exploration capabilities. It has achieved good results in benchmark test functions and engineering applications and has been verified in feature selection issues (Yu et al., 2020). The idea of introducing QRG and water circulation (WC) mechanisms in SMA was given by Cai et al. (Yu et al., 2021). Chen et al. (2020) introduced a hybrid algorithm with the combination of K-means and quantum behavior inspired by Ant Lion Optimized for data clustering and successfully applied it to intrusion detection. Deng et al. (2021) introduced quantum double-chain coding technology and quantum revolving gate into differential evolution algorithm and combined mutation strategy to further improve large-scale complex problems. The latest study by Dahi and Alba (2022) applied quantum-inspired metaheuristics to solve the Mobility Management Problem (MMP) and provides a new vision of quantum-inspired metaheuristics in conjunction with a comprehensive analysis of the quantum hardware.

In summary, this paper proposes a quantum-inspired moth-flame optimizer with enhanced local search capability (QLSMFO). The proposed algorithm combines quantum computing and the moth-flame optimizer. Quantum coding and quantum revolving gates are introduced in the initial period of the algorithm to enrich the swarm diversity as well as boost the global search capability. Then, a modified local search strategy is introduced to reinforce the mining capability. Finally, the poor solutions of the quantum moth population are selected to be updated by the Levy flight method to generate more promising solutions. The contribution of this study is primarily as follows:

(1) Quantum coding is introduced for moth swarm to enrich population diversity and further promote a more robust global search capability.
(2) The quantum revolving gate primarily balances the exploration and exploitation capabilities while guiding moths to more promising solutions and preventing them from falling into local optima.
(3) By adding an enhanced local search to improve the exploit capability and enhance the mining accuracy, moths can evade the local optimum in various ways.
(4) The proposed QLSMFO is used to solve the cluster analysis problem and has good clustering results on ten well-known UCI datasets.

The rest of this study is structured as outlined below. Section 2 fully elaborates on the problem of cluster analysis. An overview of the standard MFO algorithm in Section 3. Section 4 illustrates the specific improvement strategies of the QLSMFO. Section 5 carries out simulation experiments and analysis of results. Finally, conclusions and future work are available in Section 6.

## 2 Clustering Problem

### 2.1 Mathematical Definition

Clustering is characterized as unsupervised learning due to the absence of labeling or grouping information for each data instance. The K-Means algorithm is a classical unsupervised clustering method, which was introduced by MacQueen (1967) and has been widely used since it was proposed. To clearly illustrate the clustering problem, suppose dataset *D* is classified into *k* different clusters. There is *n* datum in dataset *D*, and each datum has *l* attributes. So, dataset *D* can be expressed as $D=\left\{x_{1},x_{2},\ldots ,x_{n}\right\}$ , where $x_{i}=\left(d_{1},d_{2},\ldots ,d_{l}\right)$ . *k* clusters are represented by $S=\left\{S_{1},S_{2},\ldots ,S_{k}\right\}$ , each cluster $S_{i}$ corresponding to a clustering center $c_{i}\left(i=1,2,\ldots ,k\right)$ . Thus, $S\left(S_{1},S_{2},\ldots ,S_{k}\right)$ should satisfy the following conditions:

(1) $\cup_{i=1}^{k}S_{i}=D$

(2) $S_{i}\neq \emptyset ,i=1,2,\ldots ,k$

(3) $S_{i}\cap S_{j}=\emptyset ,i,j=1,2,\ldots ,K,i\neq j$

### 2.2 Clustering Criteria

Dataset *D* is grouped into *k* clusters, where each cluster $S_{i}\left(i=1,2,\ldots ,k\right)$ has one cluster center $c_{i}\left(i=1,2,\ldots ,k\right)$ . The location of the center has a great impact on the clustering effect, so determining the center vector is a very important key point. Clustering requires the similarity or distance of sample features as the basis for whether they belong to a certain class. Then, the samples that are similar are grouped into one class, and those that are not grouped into one class. There are several ways to measure the similarity or distance of sample features. Several typical similarity metrics are the Minkowski distance (Gan et al., 2020) (Manhattan distance (Xu and WunschII, 2005), Euclidean distance (Jianchang Mao and Jain, 1996) and Chebyshev distance), Mahalanobis distance, and cosine similarity (Xu and WunschII, 2005), etc. In clustering, using different ways to measure similarity may yield different results, so it is very important to choose an appropriate distance or similarity when clustering. Since the Euclidean distance is relatively simple and basically reflects the effect of the clustering problem. Therefore, the Euclidean distance is used as the criterion for evaluating the clustering effect in this paper, and its definition is as follows:

$$d\left(x_{i},c_{j}\right)=\sqrt{\sum_{m=1}^{l}{\left(x_{im}-c_{jm}\right)}^{2}}$$ (1)

where *l* indicates the number of attributes for each data, $x_{i}\left(i=1,2,\ldots ,n\right)$ represents the *i*-th data in dataset *D*, $c_{j}\left(j=1,2,\ldots ,k\right)$ and is the *j*-th clustering center. The distance from data $x_{i}$ to the cluster center $c_{j}$ is calculated using Eq. 1 to determine which cluster the data belongs to. If $d\left(x_{i},c_{best}\right)<d\left(x_{i},c_{others}\right)$ , $c_{best}$ is a certain center belonging to $c_{j}\left(j=1,2,\ldots ,k\right)$ . $c_{others}$ represents other cluster centers exclusive of $c_{best}$ , then we assign the data $x_{i}$ to the cluster $S_{best}$ .

### 2.3 The Objective Function of Clustering

This paper presents the QLSMFO algorithm to settle the cluster analysis problem. With the aim of clearly describing the evaluation process, suppose there is a dataset $D=\left\{x_{1},x_{2},\ldots ,x_{n}\right\}$ divided into *k* clusters, where each data holds *l* attributes and can be expressed as $x_{i}=\left(d_{1},d_{2},\ldots ,d_{l}\right)$ . The aim of clustering is to find the location of the *k* centers corresponding to the *k* clusters of the dataset, in such a way that all data are grouped into the clusters to which they belong. It is necessary to find the optimal location of the cluster center, the solution should be structured as a one-dimensional vector of length k×l . The individual in the QLSMFO algorithm denotes the coordinate vectors of the k cluster centers, and each moth is defined as $C={\left\{c_{1},c_{2},\ldots ,c\right\}}_{k}$ . The objective function adopted in this paper is the sum of the intra-cluster distances (SIDC) (Gonzalez, 1985). It is commonly used as criteria to judge a good classification. A smaller value of SICD indicates better clustering. Therefore, the objective function is to minimize the SICD, as shown in Eq. 2:

$$f\left(D,C\right)=\sum_{i=1}^{n}\min\left\{\left\|x_{i},c_{j}\right\||j=1,2,\ldots ,k\right\}$$ (2)

where *D* refers to the given dataset, and *C* is the set of cluster centers.

## 3 Moth-Flame Optimizer

### 3.1 Inspiration

The MFO is inspired by the phenomenon of moth jumping on fire. The reason behind this phenomenon is a navigation method called transverse orientation of moths in nature. Moths flying at a constant angle to the moonlight are able to fly in a straight line and at the shortest distance to save energy. However, artificial light at night is troublesome for them. The light emitted from an artificial light source is a ray centered on the light source. If the moths still fly at a fixed angle to the light, they will fly to the center of the light source in a spiral trajectory.

### 3.2 Mathematical Model

The MFO algorithm establishes a mathematical model for the spiral flight of moths around flames. Moths and flames represent candidate solvers within the search space. But their location is updated in a different way. The moths' population is described by a matrix *M*. A one-dimensional array *OM* was used to store the fitness values calculated for all the moths. As shown below.

$$M=\left[\begin{array}{cccc} m_{11} & m_{12} & \cdots & m_{1d}\\ m_{21} & m_{22} & \cdots & m_{2d}\\ \vdots & \vdots & \ddots & \vdots \\ m_{n1} & m_{n2} & \cdots & m_{nd} \end{array}\right],\;OM=\left[\begin{array}{c} OM_{1}\\ OM_{2}\\ \vdots \\ OM_{n} \end{array}\right]$$ (3)

where *d* refers to the problem dimension, *n* denotes the size of the moth swarm.

The definition of the flame is another critical part, using a matrix *F* which stores information about the position of the flame. The values of the objective function corresponding to all flames are recorded in a one-dimensional array *OF* as shown in Eq. 4:

$$F=\left[\begin{array}{cccc} f_{11} & f_{12} & \cdots & f_{1d}\\ f_{21} & f_{22} & \cdots & f_{2d}\\ \vdots & \vdots & \ddots & \vdots \\ f_{n1} & f_{n2} & \cdots & f_{nd} \end{array}\right],\;OF=\left[\begin{array}{c} OF_{1}\\ OF_{2}\\ \vdots \\ OF_{n} \end{array}\right]$$ (4)

The spiral trajectory of the moth flying around the flame is described by a mathematical model expressed by Eq. 5:

$$M_{i}=S\left(M_{i},F_{j}\right)$$ (5)

where S(⋅) refers to the spiral trajectory of the moth around the flame. $M_{i}$ and $F_{j}$ , respectively, denote the *i*-th moth and the *j*-th flame.

$$S\left(M_{i},F_{j}\right)=D_{i}\cdot e^{bt}\cdot \cos\left(2\pi t\right)+F_{j}$$ (6)

where *b* determines the spiral shape and is set to 1, *t* indicates a random number between [*r*, 1]. In addition, *r* denotes a linearly decreasing function with a change interval of the value domain from −1 to −2. The function is shown below:

$$r\left(it\right)=-1-\frac{it}{Max\_it}$$ (7)

where *it* denotes the current generation, *Max_it* denotes the largest number of generations. $D_{i}$ is the distance from the *i*-th moth to the *j*-th flame and is calculated by Eq. 8:

$$D_{i}=\left|F_{j}-M_{i}\right|$$ (8)

The position of the flame is obtained by the moths ordered in accordance with the fitness value, then the individual moth regenerates its location in accordance with the respective flame using Eq. 6. Although this position update mechanism expands the search space and enhances exploration, all moths may have difficulty finding optimal solutions based on their respective flame update positions. To overcome this deficiency, a scheme for adaptively changing the number of flames is suggested and updated using Eq. 9:

$$N_{f}=round\left(N-it\times \frac{N-1}{Max\_it}\right)$$ (9)

where *N* represents the maximal size of the flames swarm.

**FIGURE 1:**
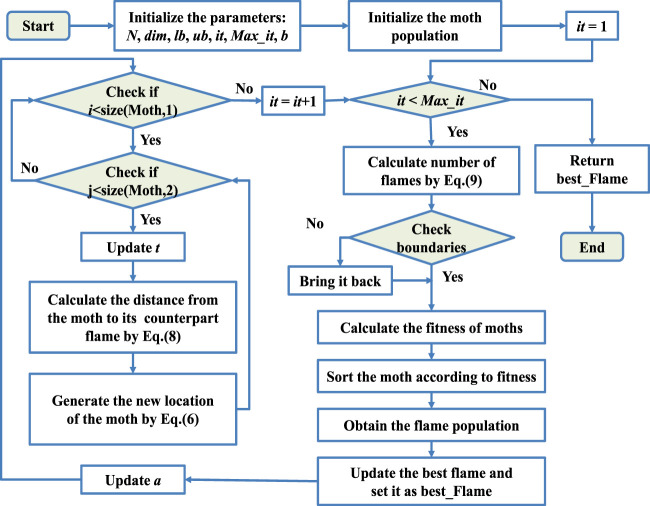
Flowchart of MFO.

The pseudo-code of the MFO is shown in Algorithm 1, and the flow chart is expressed in Figure 1.

Algorithm 1 Pseudo-code of MFO.

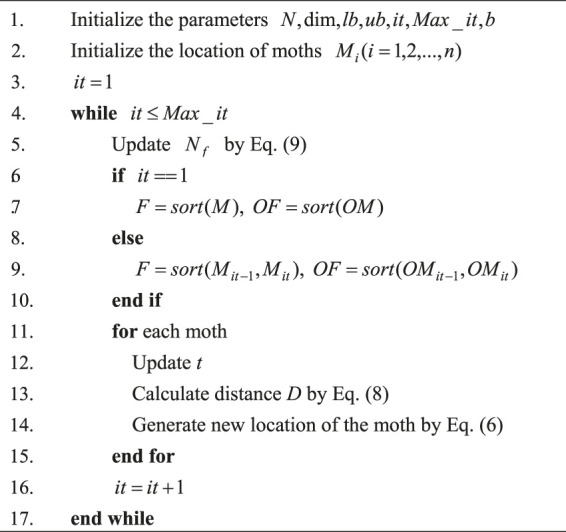

## 4 Quantum-Inspired Moth-Flame Optimizer With Enhanced Local Search Strategy

Due to the advantages of fast convergence speed and simple overall structure, the MFO algorithm is frequently employed in real-life problems, and the results obtained are competitive. However, when solving high-dimensional, multi-constraint complex problems, the convergence of the MFO algorithm turns out to be slower and frequently drops into local optima. To overcome these shortcomings, three strategies are introduced in the standard MFO algorithm. 1) Quantum coding and quantum revolving gate. 2) An improved local search based on SFLA (Eusuff et al., 2006). 3) Levy flight. The remainder of Section 4 describes in detail the contents of these three improvement strategies.

### 4.1 Quantum-Inspired Based MFO

#### 4.1.1 Quantum Encoding

In quantum computing, the minimum unit to store information is to be called a quantum bit (qubit). Distinguished from a memory cell (bit) in a classical computer, a qubit can be a superposition of “1” state and “0” state. The definition of a qubit is given in the following:

|φ〉=α|0〉+β|1〉 (10)

where α and β are two complex numbers, $\text{|}\alpha {\text{|}}^{2}$ and $\text{|}\beta {\text{|}}^{2}$ represent the probability amplitudes of the “0” state and the “1” state, respectively. And they satisfy the relation $\text{|}\alpha {\text{|}}^{2}+\text{|}\beta {\text{|}}^{2}=1$ . In order to make the equation constant, a qubit can be expressed as Eq. 11.

$$|\varphi \rangle =\left[\begin{array}{c} \alpha \\ \beta \end{array}\right]=\left[\begin{array}{c} \cos\left(\theta \right)\\ \sin\left(\theta \right) \end{array}\right],\theta \in \left[0,2\pi \right]$$ (11)

In the quantum-inspired moth-flame optimization algorithm, the individual quantum moths (*QM*) are represented as follows:

$$QM_{i}=\left(\varphi_{1},\varphi_{2},\ldots ,\varphi_{d}\right)=\left(\begin{array}{c} \cos\left(\theta_{i1}\right),\cos\left(\theta_{i2}\right),\ldots ,\cos\left(\theta_{id}\right)\\ \sin\left(\theta_{i1}\right),\sin\left(\theta_{i2}\right),\ldots ,\sin\left(\theta_{id}\right) \end{array}\right)$$ (12)

where $QM_{i}$ denotes the location of the *i*-th moth, $\theta_{ij}\in \left(0,2\pi \right),\;1\leq i\leq n,\;1\leq j\leq d$ , *n* represents the number of moths in the population, and *d* denotes the dimension of a qubit. Each quantum moth occupies two locations in the search space, and each location stands for a candidate solution to the problem, which is respectively defined as shown in Eqs 13 and 14:

$$QM_{ic}=\left(\cos\left(\theta_{i1}\right),\cos\left(\theta_{i2}\right),\ldots ,\cos\left(\theta_{id}\right)\right)$$ (13)

$$QM_{is}=\left(\sin(\left(\theta_{i1}\right),\sin\left(\theta_{i2}\right),\ldots ,\sin\left(\theta_{id}\right)\right)$$ (14)

#### 4.1.2 Quantum Initialization

Step 1: Initialize angle matrix

The moth population contains *N* individuals, and the problem dimension is *dim*. The probability amplitude is used to represent the state of a qubit and it is generated according to the angle matrix. When carrying out quantum initialization, it is necessary to establish an angle matrix of *N* * *dim*, and the search range of angle is 0 to 2π .

$$\theta_{ij}=lb_{ij}+rand\left(0,1\right)\cdot \left(ub_{ij}-lb_{ij}\right),\;1\leq i\leq n,\;1\leq j\leq d$$ (15)

where $lb_{j}$ and $ub_{j}$ indicates the minimum and maximum boundaries for *j*-th the dimension of the problem, and *rand* (0,1) is a number generated randomly between 0 and 1. The value of $lb_{j}$ and $ub_{j}$ are set to 0 and 2π , respectively. The initialized angle matrix is shown below:

$$\theta =\left[\begin{array}{cccc} \theta_{11} & \theta_{12} & \cdots & \theta_{1d}\\ \theta_{21} & \theta_{22} & \cdots & \theta_{2d}\\ \vdots & \vdots & \ddots & \vdots \\ \theta_{n1} & \theta_{n2} & \cdots & \theta_{nd} \end{array}\right]$$ (16)

Step 2: Initialize quantum population

*QM* represents a quantum moth matrix containing *N* quantum moths, each quantum moth occupying two positions in the search space, each position representing a candidate solution to the problem. The expression for the quantum population is as follows:

$$QM=\left[\begin{array}{c} QM_{1}\\ QM_{2}\\ \vdots \\ QM_{n} \end{array}\right]=\left[\begin{array}{c} QM_{1c}\\ QM_{1s}\\ QM_{2c}\\ QM_{2s}\\ \vdots \\ QM_{nc}\\ QM_{ns} \end{array}\right]=\left[\begin{array}{cccc} \cos\left(\theta_{11}\right) & \cos\left(\theta_{12}\right) & \cdots & \cos\left(\theta_{1d}\right)\\ \sin\left(\theta_{11}\right) & \sin\left(\theta_{12}\right) & \cdots & \sin\left(\theta_{1d}\right)\\ \cos\left(\theta_{21}\right) & \cos\left(\theta_{22}\right) & \cdots & \cos\left(\theta_{2d}\right)\\ \sin\left(\theta_{21}\right) & \sin\left(\theta_{22}\right) & \cdots & \sin\left(\theta_{2d}\right)\\ \vdots & \vdots & \ddots & \vdots \\ \cos\left(\theta_{n1}\right) & \cos\left(\theta_{n2}\right) & \cdots & \cos\left(\theta_{nd}\right)\\ \sin\left(\theta_{n1}\right) & \sin\left(\theta_{n2}\right) & \cdots & \sin\left(\theta_{nd}\right) \end{array}\right]$$ (17)

If it is necessary to calculate the fitness value to evaluate the individual quality, it needs to be carried out after solution space conversion. This part will be described in detail in Section 4.1.4.

#### 4.1.3 Quantum Rotation Gate

**FIGURE 2:**
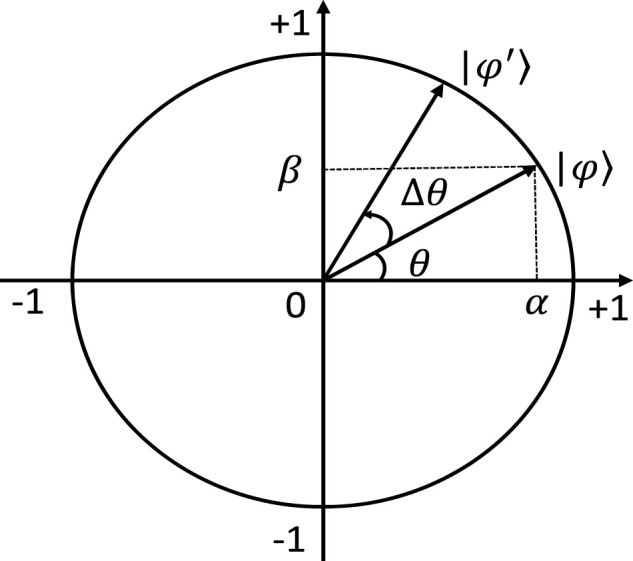
Quantum rotation angle updating.

In quantum computing, quantum operators are used to manipulating a quantum to change the relative phase of the quantum. The trade-off between global and local search is implemented by adjusting the rotation angle and direction of the QRG. Moreover, Figure 2 illustrates the position change of the QRG before and after changing the rotation angle. In QLSMFO, the expression of the quantum revolving gate is as follows:

$$U\left(\xi \left(\Delta \theta \right)\right)=\left[\begin{array}{cc} \cos\left(\xi \left(\Delta \theta \right)\right) & -\sin\left(\xi \left(\Delta \theta \right)\right)\\ \sin\left(\xi \left(\Delta \theta \right)\right) & \cos\left(\xi \left(\Delta \theta \right)\right) \end{array}\right]$$ (18)

where ξ(⋅) is a function of the rotation angle (Δθ) , which will be described in detail later.

The new qubit is updated by using the quantum revolving gate, as shown in the following formula:

$$\left[\begin{array}{c} \alpha_{i}^{\prime }\\ \beta_{i}^{\prime } \end{array}\right]=U\cdot \left[\begin{array}{c} \alpha_{i}\\ \beta_{i} \end{array}\right]$$ (19)

In QLSMFO, instead of using the traditional fixed angle for the rotation angle of the quantum revolving gate, the Differential Evolution (DE) algorithm is employed for dynamically updating the angle size and direction. The process of adjusting the rotation angle by the DE algorithm can be seen as under:

##### 4.1.3.1 Mutation Operation

The rotation angle $\theta_{ij}\left(1\leq i\leq n,\;1\leq j\leq d\right)$ in each quantum moth $QM_{i}$ is updated using the DE/rand/1 strategy using the following formula:

$$v_{ij}=\theta_{r_{1}j}+F\left(\theta_{r_{2}j}-\theta_{r_{3}j}\right)$$ (20)

where $r_{1}$ , $r_{2}$ , and $r_{3}$ are random integers between [1, *d*].

##### 4.1.3.2 Crossover Operation

The new angle $u_{ij}$ and the previous angle $\theta_{ij}$ are crossed with a certain probability, and the crossover operation is shown in Eq. 21.

$$u_{ij}=\{\begin{array}{cc} v_{ij}, & rand\leq CR\;or\;j=randi\\ \theta_{ij}, & else \end{array}$$ (21)

where *CR* represents the probability of crossover, which is a random number between [0,1]. *randi* refers to a random integer between [1, *d*].

##### 4.1.3.3 Rotation Angle Acquisition

Rotation angle $\xi \left(\Delta \theta \right)=S\left(\alpha_{i},\beta_{i}\right)\times \left|u_{ij}-\theta_{ij}\right|$ . The sign function S(⋅) represents the direction of the rotation angle and the updated formula of the sign function is as Eq. 22. $\left|u_{ij}-\theta_{ij}\right|$ represents the magnitude of the rotation angle.

$$S\left(\alpha_{i},\beta_{i}\right)=sign\left(\alpha_{i}\times \beta_{i}\right)$$ (22)

#### 4.1.4 Solution Space Transformation

The fitness value was considered to assess the quality level of each moth. It is necessary to transform the solution space of the individual’s position. Assuming that the solution space of the definition problem is $\Omega_{QLSMFO}=\left[a,b\right]$ , the conversion formula of solution space is listed in the following equations:

$$RM_{ic}=\frac{a\left(1-\alpha_{i}\right)+b\left(1+\alpha_{i}\right)}{2}$$ (23)

$$RM_{is}=\frac{a\left(1-\beta_{i}\right)+b\left(1+\beta_{i}\right)}{2}$$ (24)

### 4.2 Enhanced Local Search Strategy

To obviate the original MFO algorithm from trapping into local optima, an individual moth is designed to fly in a spiral trajectory according to the corresponding flame position instead of flying towards a single flame. While this mechanism improves the moth’s ability to fall into local optima, it also reduces the ability to mine more promising solutions. To solve this defect, the standard MFO algorithm introduces a mechanism for adjusting the number of flames in the local search stage, which enhances the probability of obtaining the optimal solution to a certain extent. However, there is still much scope for improvement in terms of convergence rate and precision.

**FIGURE 3:**
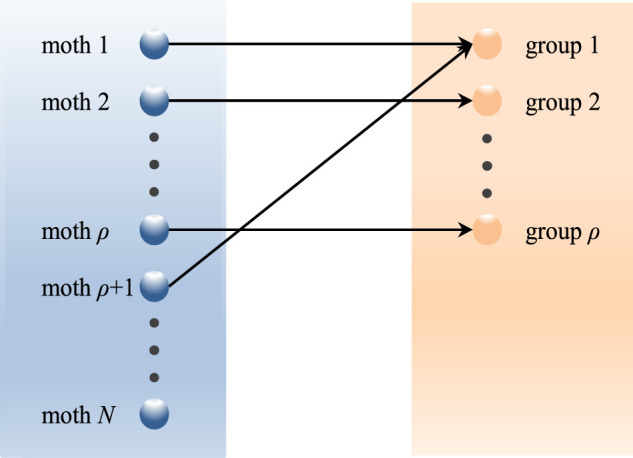
Diagram of grouping rules.

For the purpose of finding the optimal solution at a faster rate and obtaining higher accuracy, this paper adopts a boosted approach based on the standard local search strategy of SFLA. This strategy divides the moths into ρ groups according to their fitness values. Grouping rules: 1) The moth ranked first is assigned to the first group, and the moth ranked second is assigned to the second group until the moth ranked ρ -th is assigned to the ρ -th group. 2) The (ρ+1) -th moth is assigned to the first group, and the procedure is repeated till the last moth is assigned. Figure 3 shows the grouping rules.

The moths with the best fitness value and the moths with the worst fitness value in each group are defined as $M_{b}$ and $M_{w}$ , respectively. The globally optimal moth is defined as $M_{g}$ . The worst moth in each group updates its position according to the local optimum, the global optimum or both. The promising solutions are fully utilized to obtain the optimum solution for the purpose of avoiding collapsing towards a local optimum and enhancing the mining ability. The specific update steps are shown in steps 1–3. Additionally, Algorithm 2 presents the pseudo-code for the local search mechanism.

Step 1. The worst moth adjusts its position with respect to the optimal value in the group, as shown in Eq. 25.

$$M_{new}=c\cdot rand\cdot \left(M_{b}-M_{w}\right)+M_{w}$$ (25)

Step 2. If a better position cannot be obtained in Step 1, then the worst moth is updated according to the global optimum position according to Eq. 26.

$$M_{new}=c\cdot rand\cdot \left(M_{g}-M_{w}\right)+M_{w}$$ (26)

Step 3. If a better position cannot be obtained in Step 2, the worst moth uses Eq. 27 to update the position according to the optimal moth in the group and the global optimal moth.

$$M_{new}=rand\cdot \left(\left(M_{g}+M_{b}\right)/2-M_{w}\right)+M_{w}$$ (27)

According to the local search strategy in SFLA, steps 1–3 here will be repeated ζ times.

Algorithm 2 Pseudo-code of local search mechanism.

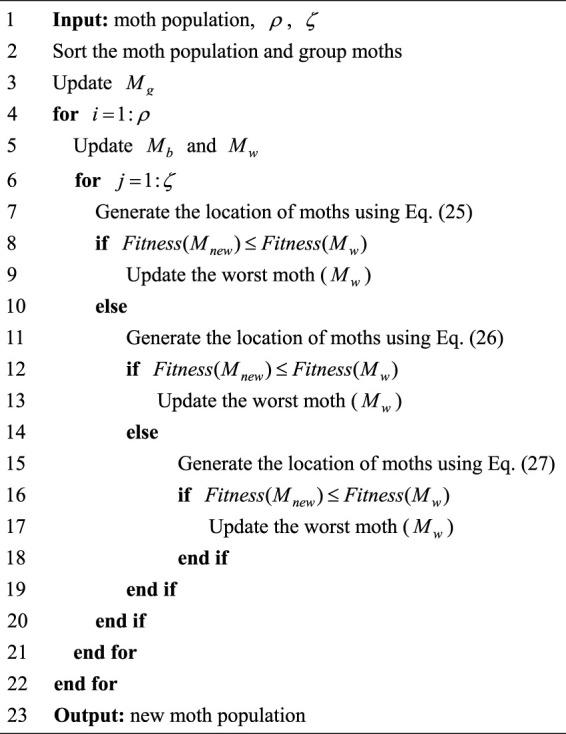

### 4.3 Poor Solution Update Using Levy Flight

After *L* iterations, moths with larger total fitness values (for the minimization problem) are defined as individuals with weak search ability, and these moths are defined as poor moths. Levy flight is introduced to update the position of poor moths to obtain more promising solutions. The update formula of the poor moths is shown in Eq. 28.

$$M_{i}^{\prime }=M_{i}\times \left(1+Levy\left(\beta \right)\right)$$ (28)

### 4.4 Description of QLSMFO Algorithm

**FIGURE 4:**
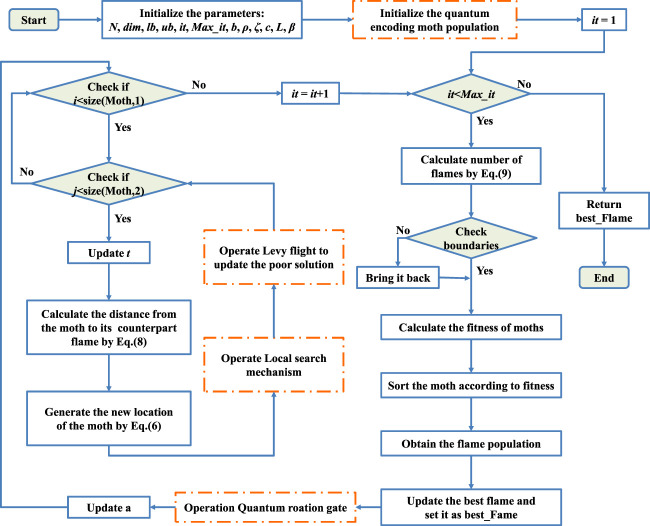
Flowchart of QLSMFO.

The pseudo-code of the QLSMFO is exhibited in Algorithm 3. Then the flowchart of the QLSMFO is exhibited in Figure 4.

Algorithm 3 Pseudo-code of QLSMFO.

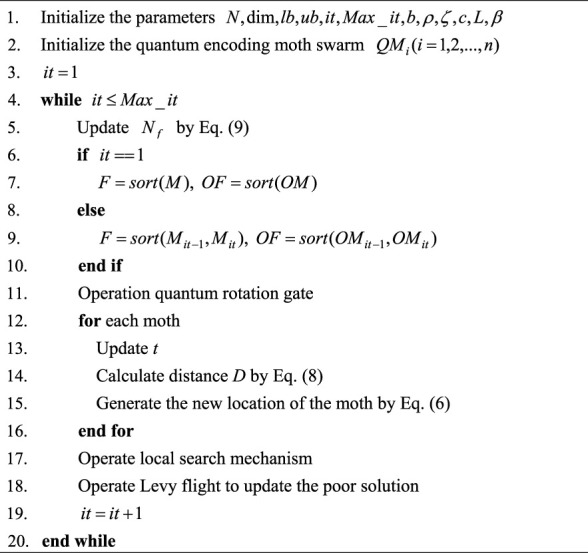

### 4.5 Computational Complexity of the QLSMFO Algorithm

In the QLSMFO algorithm, the computational cost is mainly composed of seven parts: quantum initialization, fitness value calculation, generating flame population, QRG operation, moth position update, local search strategy of SFLA, and Levy flight. Here *n* is the number of moth individuals and *d* is the dimension. In the quantum initialization phase, first, use the function rand to generate an n*d matrix between 0 and 2π . The time complexity of this operation is *O* (*nd*). Then it is converted into a quantum population according to the angle matrix, and the time complexity is *O* (*nd*). After entering the loop, the computational complexity is also related to the number of iterations *t*. In the iterative phase, the computational cost of fitness value calculation, generating flame population, QRG operation, and moth position update are all *O*(*t*) *O* (*nd*). The computational complexity of the local search stage is O(t)O(ρζ) , and ρζ is less than $n^{2}$ . In the final Levy flight stage, the computational complexity is O(t)O(nL) , and *L* is less than *n*.

The time complexity of QLSMFO is the sum of the time complexity of the above seven parts, as shown below:

$$\begin{array}{l} T=O\left(nd\right)+O\left(t\right)O\left(nd\right)+O\left(t\right)O\left(nd\right)+O\left(t\right)O\left(nd\right)\\ +O\left(t\right)O\left(nd\right)+O\left(t\right)O\left(n^{2}\right)+O\left(t\right)O\left(n^{2}\right)\\ =O\left(nt\left(n+d\right)\right) \end{array}$$ (29)

The time complexity of the original MFO algorithm is O(MFO)=O(nt(n+d)) (Mirjalili, 2015a). The computational complexity of the proposed algorithm is the same as the original MFO algorithm.

## 5 Experiment Results

All experiments in this paper were implemented on MATLAB R2018(b), running on a desktop computer with Windows 10 operating system, Intel(R) Core(TM) i7-9700 CPU, running frequency of 3.00 GHz and 16 GB of memory.

### 5.1 Parameter Settings

With the purpose of validating the performance of QLSMFO, the improved algorithm is compared with K-means (MacQueen, 1967) and thirteen current mainstream optimization algorithms. They are the artificial bee colony (ABC) algorithm (Karaboga and Basturk, 2007), ant lion optimizer (ALO) (Mirjalili, 2015b), cuckoo search (CS) algorithm (Yang and Deb, 2009), DE (Storn and Price, 1997), flower pollination algorithm (FPA) (Yang, 2012), GWO (Mirjalili et al., 2014), Moth-flame optimizer (MFO) (Mirjalili, 2015a), multi-verse optimizer (MVO) (Mirjalili et al., 2016), PSO (Kennedy and Eberhart, 1995), whale optimization algorithm (WOA) (Mirjalili and Lewis, 2016), SFLA (Eusuff et al., 2006), quantum encoding bat algorithm (QBA) (Luo et al., 2017), Gaussian quantum behaved particle swarm optimization (GQPSO) (Coelho, 2010). The parameter settings of the above-mentioned comparison algorithms are reported in Table 1.

**TABLE 1:** Parameter value setting for the comparison algorithms.

Algorithms	Parameter values
ABC	Limit=5
ALO	*NAN*
CS	$p_{a}=0.25$
DE	F=0.5,CR=0.9
FPA	p=0.8
GWO	a∈[2,0]
MFO	b=1,t∈[r,1],r∈[−1,−2]
MVO	$WEP_{\min}=0.2,WEP_{\max}=1$
PSO	$w=0.7298,C_{1}=C_{2}=1.4962$
WOA	b=1,t∈[r,1],r∈[−1,−2]
SFLA	*Memeplex Size* = 10, *Number of Memeplexes* = 5
QBA	*alpha* = 0.95,*gamma* = 0.05
GQPSO	$w_{1}=0.5,w_{2}=1,C_{1}=C_{2}=1.5$

The largest value of generations for each algorithm is *Max_it* = 200, and the number of moth swarm is *N* = 50. The dimensions are the same as the number of attributes in the benchmark dataset. The datasets used in this paper include two artificial datasets and eight UCI classic datasets. The specific characteristics of the dataset will be further introduced in Section 5.2. All algorithms will be independently executed 20 times.

### 5.2 Datasets

Among the 10 benchmark datasets, Artificial Datasets I and II are artificial datasets selected from the literature (Niknam and Amiri, 2010), and the remaining 8 datasets are related to life and physics from UCI. Table 2 summarizes the number of attributes, clusters, and instances and the application areas of ten benchmark datasets.

**TABLE 2:** Details of the ten clustering benchmark datasets.

No.	Datasets	Attributes	Clusters	instances	Area	References
1	Artificial Dataset I	3	5	250	Numerical	Niknam and Amiri (2010)
2	Artificial Dataset II	2	4	600	Numerical	Niknam and Amiri (2010)
3	Iris	4	3	150	Life	Frank and Asuncion (2010)
4	Glass	9	6	214	Physical	Frank and Asuncion (2010)
5	Wine	13	3	178	Physical	Frank and Asuncion (2010)
6	Breaster cancer Wisconsin (Original)	9	2	683	Life	Frank and Asuncion (2010)
7	CMC	9	3	1,473	Life	Frank and Asuncion (2010)
8	Seeds	7	3	210	Life	Frank and Asuncion (2010)
9	Statlog (Heart)	13	2	270	Life	Frank and Asuncion (2010)
10	Haberman’s survival	3	2	306	Life	Frank and Asuncion (2010)

### 5.3 Results Analysis

Table 3 reports the statistical results of the experiments performed by QLSMFO and K-Means algorithms and other thirteen metaheuristics algorithms on ten test datasets. The data in the table are presented in the form of four decimal places, except for Std., which uses scientific notation to retain two decimal places. In the table, Best indicates optimal fitness value, Worst indicates the worst fitness value, Mean indicates average fitness value, and Std. indicates standard deviation. The four indicators are the statistics obtained by each algorithm in 20 independent runs. Friedman test is applied to the four indicators in Table 3. The penultimate column FAR indicates the Friedman’s average ranking, and the last column Rank indicates the final ranking. It can be observed through Table 3, that compared with other comparison algorithms, QLSMFO ranks the best on the four indicators (Best, Worst, Mean, and Std.) on all datasets except the seed dataset. QLSMFO achieves second place in the Std. index on the seed dataset. These data show that QLSMFO possesses excellent precision and reliability.

**TABLE 3:** Simulation results for clustering algorithm after 20 runs on 10 datasets.

Algorithms	Indicators	Art 1	Art 2	Iris	Glass	Wine	Cancer	CMC	Seeds	Heart	Survival	FAR	Rank
K-means	Best	1,720.7628	514.6614	97.1233	215.4263	16,555.6794	2,984.7454	5,542.8731	313.3424	10,695.7974	2,625.1076	10.6	11
	Worst	2,483.8590	899.7352	123.6660	255.0263	18,436.9521	2,991.2629	5,546.3438	315.1928	10,700.8385	3,196.5920	7.5	6
	Mean	2,182.7909	591.9153	106.5132	228.4885	17,177.6946	2,988.2538	5,544.2166	314.0933	10,697.8800	2,656.4600	8.8	10
	Std.	3.4954E+02	1.5776E+02	1.2069E+01	1.4471E+01	8.7556E+02	2.5164E+00	1.1311E+00	6.1125E-01	2.4954E+00	1.2715E+02	8.5	10
ABC	Best	2,621.0031	534.2840	97.2462	357.5500	16,435.6636	2,991.9163	5,773.0571	316.9397	10,645.0502	2,566.9889	11.9	13
	Worst	3,240.0348	615.5024	124.6974	410.9385	16,743.6069	4,480.4801	6,099.3676	388.1249	10,720.3553	2,567.0281	9.5	11
	Mean	2,902.6014	567.1584	104.9606	399.4768	16,569.2437	3,382.5750	5,929.5165	345.0643	10,669.4992	2,566.9915	10.6	12
	Std.	1.6420E+02	2.3308E+01	7.1803E+00	1.5803E+01	9.3189E+01	3.8740E+02	9.8639E+01	1.7575E+01	2.0286E+01	8.6369E-03	7.8	5.5
ALO	Best	1,718.2539	513.9017	96.6556	299.8880	16,318.0213	2,965.6134	5,626.9893	311.9358	10,629.3649	2,566.9889	6.6	7
	Worst	2,444.7418	908.8921	127.6677	392.3352	16,401.9475	3,099.4506	5,923.8279	330.4222	10,706.0674	2,567.8248	7.6	7
	Mean	2,204.0973	572.3286	99.8589	339.7677	16,352.6888	2,993.9541	5,760.3530	313.8693	10,659.0977	2,567.0725	7.5	7.5
	Std.	3.2747E+02	1.4273E+02	9.5129E+00	2.9350E+01	2.5321E+01	3.7799E+01	9.0739E+01	4.0061E+00	2.1123E+01	2.5730E-01	8	7
CS	Best	1,722.4942	513.9018	96.6573	249.3329	16,296.0299	2,964.4719	5,541.6300	311.9462	10,623.3962	2,566.9889	6.3	6
	Worst	2,276.0927	514.3841	97.5704	282.7527	16,303.9573	2,967.0588	5,573.5388	314.0035	10,625.2040	2,566.9889	3.2	2
	Mean	1,828.3900	513.9708	96.8449	267.7335	16,299.2245	2,964.9179	5,549.8029	312.6272	10,623.8756	2,566.9889	3.2	2
	Std.	1.3254E+02	1.1914E-01	2.5819E-01	9.6864E+00	1.9661E+00	5.7000E-01	8.9369E+00	5.4122E-01	4.8201E-01	5.4630E-07	3.1	2
DE	Best	1,718.4065	513.9017	96.6555	308.6971	16,345.1128	2,974.3611	5,539.5190	311.9595	10,623.3159	2,566.9889	7	8
	Worst	2,468.3549	516.9062	120.7318	422.9514	17,659.3237	3,109.9039	5,656.5296	317.0757	11,379.4120	2,569.1538	8	8
	Mean	1,967.7027	514.2086	98.3375	352.3671	16,697.5468	3,031.1791	5,561.8980	313.8235	10,668.2034	2,567.6156	6.9	6
	Std.	3.2974E+02	7.0254E-01	5.3026E+00	3.3584E+01	3.6771E+02	4.2901E+01	2.8340E+01	1.3030E+00	1.6759E+02	8.1344E-01	8.6	11
FPA	Best	2,209.2565	553.0295	107.9338	358.2252	16,523.6271	3,125.2321	6,022.8869	357.3546	10,756.1553	2,575.2301	13.6	14
	Worst	2,590.7114	701.2459	125.3917	410.6478	16,808.5805	3,467.1155	6,415.8210	390.7098	11,375.1235	2,597.1100	10.3	12
	Mean	2,392.1632	632.2991	114.4317	390.9783	16,620.3091	3,257.2720	6,239.4660	374.3006	11,125.5227	2,585.8700	12.5	14
	Std.	1.0295E+02	3.7934E+01	4.6593E+00	1.7203E+01	7.6283E+01	8.9949E+01	1.2830E+02	1.0295E+01	1.8011E+02	6.2352E+00	8.3	9
GWO	Best	1,719.2955	514.4099	96.6967	300.8932	16,317.0858	2,964.4685	5,577.6440	312.7450	10,640.1561	2,567.2268	8.5	9
	Worst	2,420.1129	518.9048	121.4958	408.1592	16,379.2516	2,964.7198	5,881.3547	319.0629	10,679.6548	2,661.6690	5.9	3
	Mean	1,755.8602	516.3923	100.7814	350.8107	16,339.0761	2,964.5506	5,697.2697	314.2480	10,656.2902	2,596.2901	6.6	5
	Std.	1.5635E+02	1.4014E+00	8.7986E+00	2.7177E+01	1.7113E+01	7.3443E-02	9.4461E+01	1.3905E+00	1.1586E+01	3.0838E+01	6.6	3
MFO	Best	1,718.4008	513.9017	96.6556	258.1097	16,298.1081	2,964.8051	5,534.6088	311.9329	10,625.0701	2,566.9889	5.35	5
	Worst	2,701.1929	513.9017	110.7161	316.6208	16,527.7618	3,067.3649	5,972.9363	355.9924	10,701.6759	2,594.5747	6.9	5
	Mean	2,154.8576	513.9017	98.7980	277.0228	16,332.1209	2,981.9060	5,650.9171	319.2587	10,647.9703	2,568.3682	6.1	4
	Std.	3.5595E+02	1.9986E-06	4.3183E+00	1.6129E+01	5.3271E+01	2.6386E+01	1.1345E+02	1.3420E+01	2.4114E+01	6.1684E+00	7.8	5.5
MVO	Best	1,718.8952	513.9038	96.6805	312.0478	16,330.8529	2,964.7202	5,563.0206	312.2483	10,648.6368	2,567.0123	8.9	10
	Worst	3,148.8098	892.4766	125.6249	405.7594	16,452.0811	2,965.8537	5,788.7023	403.0414	10,731.3672	2,567.9309	8.1	9
	Mean	2,003.3197	532.8372	98.9310	368.3374	16,382.3227	2,965.2540	5,676.3160	317.9289	10,679.5546	2,567.2569	7.5	7.5
	Std.	4.8459E+02	8.4650E+01	6.5492E+00	2.5894E+01	3.1054E+01	3.5254E-01	5.6685E+01	2.0058E+01	2.4109E+01	3.8386E-01	8.2	8
PSO	Best	1,718.2539	513.9017	96.6555	265.2194	16,292.4155	2,964.3872	5,532.1987	311.7982	10,622.9861	2,566.9889	3.55	2
	Worst	2,444.7856	513.9017	127.6677	410.2468	16,303.2108	4,728.7901	5,533.0006	420.3489	10,623.1898	2,566.9889	6.3	4
	Mean	2,124.5283	513.9017	104.4086	299.2063	16,294.6942	3,140.8319	5,532.3505	317.2281	10,623.0399	2,566.9889	5	3
	Std.	3.4428E+02	2.0363E-08	1.3777E+01	3.1432E+01	2.5012E+00	5.4307E+02	2.2316E-01	2.4272E+01	5.5801E-02	1.3489E-08	7.3	4
WOA	Best	1,967.3942	515.1639	97.8072	314.5328	16,341.1455	2,979.3869	5,857.9515	333.4598	10,667.4260	2,567.8508	11.8	12
	Worst	3,149.4792	909.1263	130.1484	441.8749	16,509.7300	3,158.4952	6,327.4464	403.9605	11,486.1792	2,615.7618	11.9	14
	Mean	2,577.3095	614.8494	109.4386	370.4912	16,408.0293	3,015.8097	6,072.2996	366.5992	10,885.3931	2,581.9343	11.3	13
	Std.	3.1224E+02	1.6672E+02	1.2182E+01	3.5617E+01	4.5097E+01	4.0373E+01	1.3849E+02	1.8027E+01	2.3303E+02	1.4936E+01	11.1	13
SFLA	Best	1,718.2539	513.9017	96.9582	407.4744	16,394.8405	2,964.3870	5,532.1852	311.7978	10,622.9824	2,566.9889	4.5	3
	Worst	2,377.4273	862.5571	122.3652	518.1696	17,583.7551	3,454.1298	6,019.0809	311.7978	10,854.7384	2,569.1504	8.3	10
	Mean	1,912.3766	531.3345	106.1995	465.5880	16,955.6852	3,049.8002	5,583.5415	311.7978	10,637.8637	2,567.4314	7.8	9
	Std.	3.0430E+02	7.7962E+01	7.3287E+00	3.1478E+01	3.2333E+02	1.4162E+02	1.4384E+02	2.2890E-10	5.2156E+01	5.7799E-01	9.3	12
QBA	Best	1,718.2539	513.9017	96.6555	257.3020	16,320.1745	2,964.3870	5,536.2231	311.8245	10,640.7358	2,566.9889	4.9	4
	Worst	3,148.7153	908.8755	202.8310	560.1374	16,476.2419	2,964.3921	5,640.8932	420.9471	13,674.7427	3,403.4322	10.8	13
	Mean	2,649.1982	723.7572	116.0737	326.6042	16,395.7152	2,964.3872	5,567.3356	331.4431	11,252.5517	2,608.9148	10.1	11
	Std.	4.6925E+02	1.9481E+02	2.8089E+01	8.2232E+01	3.7305E+01	1.1377E-03	2.5466E+01	4.0631E+01	8.9514E+02	1.8701E+02	11.8	14
GQPSO	Best	3,564.2153	803.7830	156.8816	504.3203	17,071.9694	4,113.1492	6,854.3127	415.3774	12,468.5227	2,974.8667	15	15
	Worst	4,643.8922	984.7499	205.1782	690.7817	17,838.9329	4,562.9025	8,296.2281	549.8209	15,343.1876	3,162.2552	14.6	15
	Mean	4,167.1308	889.9289	188.9817	602.8586	17,639.4478	4,344.6041	7,564.6900	507.5001	14,138.5153	3,019.7733	15	15
	Std.	3.2538E+02	6.2621E+01	1.4246E+01	5.2994E+01	1.6473E+02	1.3509E+02	3.1965E+02	2.9398E+01	8.2092E+02	5.3796E+01	12.5	15
QLSMFO	Best	1,718.2539	513.9017	96.6555	210.5258	16,292.1870	2,964.3870	5,532.1848	311.7978	10,622.9824	2,566.9889	1.5	1
	Worst	1,718.2539	513.9017	96.6555	234.3142	16,294.3534	2,964.3870	5,532.1891	311.7978	10,622.9834	2,566.9889	1.1	1
	Mean	1,718.2539	513.9017	96.6555	220.9848	16,293.2552	2,964.3870	5,532.1852	311.7978	10,622.9825	2,566.9889	1.1	1
	Std.	5.4772E-06	1.1664E-13	1.6264E-08	9.2425E+00	7.8681E-01	2.9443E-07	9.5136E-04	9.8831E-07	2.1012E-04	5.8086E-13	1.1	1

Bold indicates the optimal value, FAR stands for Friedman’s average ranking.

**FIGURE 5:**
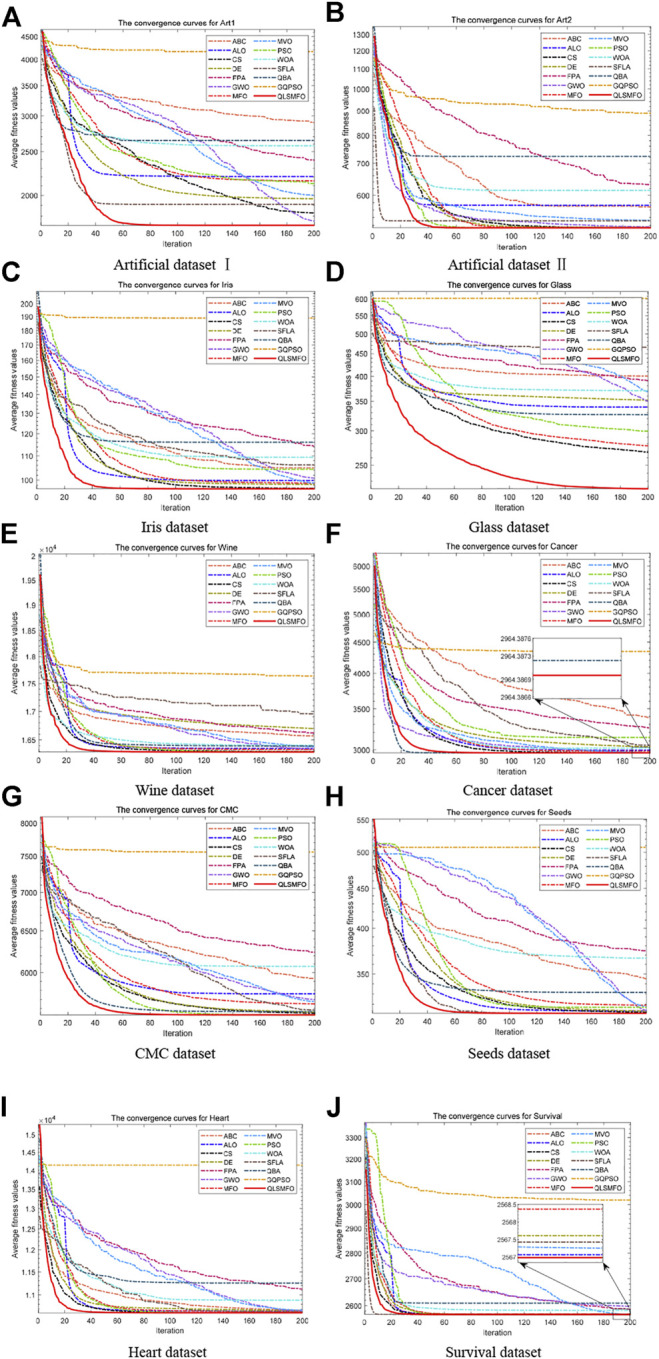
Convergence curves of all algorithms on the 10 datasets. **(A)** Artificial Dataset I. **(B)** Artificial Dataset II. **(C)** Iris dataset. **(D)** Glass dataset. **(E)** Wine dataset. **(F)** Cancer dataset. **(G)** CMC dataset. **(H)** Seeds dataset. **(I)** Heart dataset. **(J)** Survival dataset.

**FIGURE 6:**
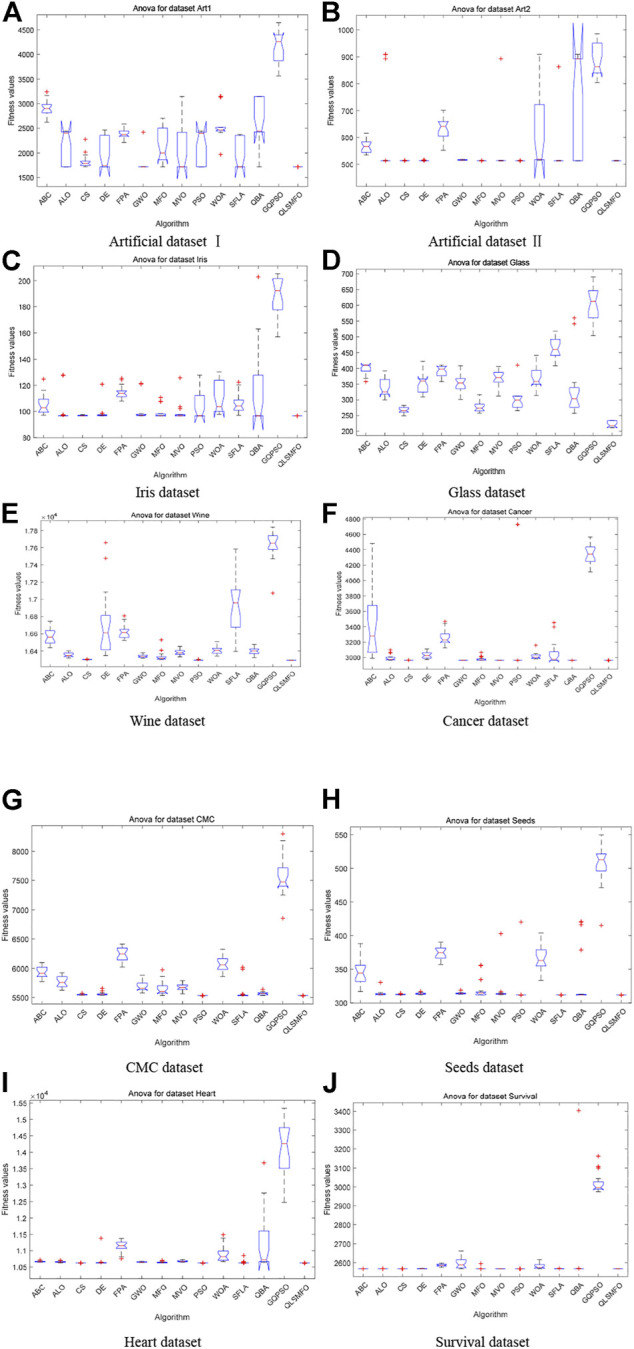
ANOVA simulation results of all algorithms on the 10 datasets. **(A)** Artificial Dataset I. **(B)** Artificial Dataset II. **(C)** Iris dataset. **(D)** Glass dataset. **(E)** Wine dataset. **(F)** Cancer dataset. **(G)** CMC dataset. **(H)** Seeds dataset. **(I)** Heart dataset. **(J)** Survival dataset.

The convergence curves of all algorithms on the 13 datasets are presented in Figure 5. The data of the curves are the average fitness values calculated by running each algorithm 20 times independently with 200 iterations. Figure 6 shows the ANOVA plot. A low median, few outliers, and a narrow height in the variance plot indicate better stability. It is clear and obvious from Figures 5 and 6 that QLSMFO possesses the advantages of rapid convergence, excellent accuracy, and outstanding stability. The specific analysis of each dataset is shown below.

#### 5.3.1 Artificial Dataset I

For Artificial Dataset I, QLSMFO won first place in all algorithms in the four indicators. ALO, PSO, SFLA, and QBA reach the same level as QLSMFO at the optimal value. It is clearly observed that the worst value of QLSMFO outperforms the optimal results derived from other algorithms. It revealed that QLSMFO has high precision and good algorithm performance. Figure 5A shows that the speed of the convergence curve of QLSMFO decreases significantly more quickly when compared with other methods. The standard deviation of QLSMFO from Table 3 is better than all algorithms by eight orders of magnitude. It can also be observed in Figure 6A that the graph of the improved algorithm is the narrowest and the median line is the lowest, indicating that the algorithm has high robustness.

#### 5.3.2 Artificial Dataset II

QLSMFO achieves the best performance on Best, Worst, Mean, and Std. Table 3 shows that ALO, DE, SFLA, and QBA is the same as QLSMFO on the best indicator. Then MFO and PSO have the same good performance as QLSMFO on Best, Worst, and Mean indicators. However, the standard deviation of QLSMFO is superior to theirs, which is seven and five orders of magnitude better than MFO and PSO, respectively. From the convergence curve in Figure 5B, although the proposed algorithm fails to converge as rapidly as SFLA in the early iterations, after 30 generations, QLSMFO converges significantly quicker than the other algorithms and is ultimately the best in accuracy. Compared with other algorithms in Figure 6B, QLSMFO has no outliers, and the box is the narrowest, so the stability of the algorithm is better.

#### 5.3.3 Iris Dataset

QLSMFO achieved the greatest performance on Best, Worst, Mean, and Std., except PSO, DE, and QBA. They achieve the same results as QLSMFO in Best indicators. However, the values of Worst, Mean, and Std. are not as good as the results of QLSMFO. From Figure 5C, it can be seen that DE has a small number of outliers, and it can be seen from the box shape that the variance of PSO is large. QLSMFO has the smallest variance and no outliers, indicating that the algorithm has high stability. Table 3 reflects that QLSMFO outperforms the other algorithms by at least eight orders of magnitude in terms of Std. indicators, which shows that the algorithm is robust.

#### 5.3.4 Glass Dataset

QLSMFO acquired the optimum value on Best, Worst, Mean, and Std. among eleven algorithms. The worst value of QLSMFO outperforms the optimal values of the other algorithms except for the K-means algorithm. In Figure 5D, it is clearly visible that QLSMFO has the fastest rate of convergence, and the final convergence accuracy has obvious advantages over other algorithms. In Table 3, the Std. index of QLSMFO has no obvious advantage compared with other algorithms, but in Figure 6D, it can be seen that the height of the box of QLSMFO is lower than that of other algorithms, indicating great robustness.

#### 5.3.5 Wine Dataset

Table 3 displays that QLSMFO obtains the optimum value on Best, Worst, Mean, and Std. The figures for PSO are very close to those of QLSMFO in terms of optimal and average values. However, it is observed from Figure 5E that QLSMFO converges faster. The higher stability of QLSMFO than PSO is observed in Figure 6E.

#### 5.3.6 Cancer Dataset

It is shown that the values of Best, Worst, Mean, and Std. of QLSMFO are optimum in Table 3. The Best indicators of CS and PSO are close to QLSMFO, at the same time, SFLA and QBA reach the same level as QLSMFO at the optimal value, but the other three indicators are not as good as the improved algorithm. QLSMFO has a significant improvement on Std. metrics, at least five orders of magnitude of excellence over other algorithms. It is observed that the worst value obtained by QLSMFO is superior to the optimum value derived from comparison algorithms. The convergence curve of QLSMFO is displayed in Figure 6F, although it is not as fast as QBA in the initial 20 generations of iteration, the convergence rate in the later period is the fastest among all algorithms.

#### 5.3.7 CMC Dataset

It is observed in Table 3 that QLSMFO achieves the best performance for all metrics compared with the comparative algorithms. Although the performance of PSO is close to that of QLSMFO, the data provided in Table 3 demonstrates that the Std. index of QLSMFO is three orders of magnitude superior to PSO, and QLSMFO has higher stability and robustness. Figure 6G shows that the convergence rate of QLSMFO decreases quicker than other methods.

#### 5.3.8 Seeds Dataset

Table 3 shows that QLSMFO all obtained the optimal values on Best, Worst, and Mean except for Std. The performance of SFLA reaches the same level as QLSMFO, although the stability of QLSMFO is not as good as that of SFLA, the gap between them is not big. And the convergence speed of QLSMFO is significantly faster than that of SFLA. Moreover, the value of its Std. the indicator is at least six orders of magnitude outperformed by other algorithms. It appears from Figure 6H that the stability of QLSMFO is higher. The convergence curve of QLSMFO drops the fastest, and the accuracy at the end of the iteration is the highest, as can be observed from Figure 5H.

#### 5.3.9 Heart Dataset

The data in Table 3 shows that QLSMFO achieves optimal values for Best, Worst, Mean, and Std. From Figure 6I, the heights of the boxes of CS, PSO, and QLSMFO are all short, indicating high stability, but the position of QLSMFO is lower, so the robustness of QLSMFO is higher than that of CS and PSO.

#### 5.3.10 Survival Dataset

QLSMFO attained the best performance in Best, Worst, Mean, and Std. from Table 3. Best metric of ABC, ALO, CS, DE, MFO, PSO, SFLA, and QBA all reach the same level as QLSMFO, and the worst and average values of CS and PSO are the same as QLSMFO. However, the standard deviation of QLSMFO is five to six orders of magnitude preferred over CS and PSO. As seen in Figure 6J that CS and PSO have outliers, but QLSMFO does not. So the stability of QLSMFO is higher than these two algorithms. From Figure 5J, although SFLA has the fastest convergence speed in the early stage, the final accuracy is not as high as that of QLSMFO.

### 5.4 Wilcoxon’s Non-Parametric Test

The Wilcoxon rank-sum test is a non-parametric statistical technique that is introduced in this research to accurately validate the experimental results of this investigation and to verify that the effect of QLSMFO is statistically significant and does not occur by coincidence. The twenty best fitness values yielded in twenty independent runs of each method were used in Wilcoxon’s non-parametric statistical test. The *p*-value shown in Table 4 is calculated from the eleven pairs of data through the Wilcoxon rank-sum test. In this study, fourteen pairs of data are formed by QLSMFO vs. ABC, QLSMFO vs. ALO, QLSMFO vs. CS, QLSMFO vs. DE, QLSMFO vs. FPA, QLSMFO vs. GWO, QLSMFO vs. MFO, QLSMFO vs. MVO, QLSMFO vs. PSO, QLSMFO vs. WOA, QLSMFO vs. SFLA, QLSMFO vs. QBA, QLSMFO vs. GQPSO and QLSMFO vs. K-means. If p≤0.05 means that the null hypothesis does not hold, indicating that there is a significant difference between the algorithms. In Table 4, *p*-values are all less than 0.05, except that SFLA has a *p*-value of 0.572 in the Survival dataset and QBA has a *p*-value of 0.273 in the Cancer dataset. These results show that the proposed algorithms have statistically significant differences.

**TABLE 4:** *p*-values generated by Wilcoxon rank-sum test.

Datasets				QLSMFO vs.										
	ABC	ALO	CS	DE	FPA	GWO	MFO	MVO	PSO	WOA	SFLA	QBA	GQPSO	K-means
Art I	6.80E-08	6.80E-08	6.80E-08	6.80E-08	6.80E-08	6.80E-08	6.80E-08	6.80E-08	6.80E-08	6.80E-08	3.15E-02	9.17E-08	6.80E-08	6.28E-08
Art II	5.52E-08	5.52E-08	5.52E-08	5.52E-08	5.52E-08	5.52E-08	5.52E-08	5.52E-08	5.52E-08	5.52E-08	5.71E-03	5.52E-08	5.52E-08	5.48E-08
Iris	6.72E-08	6.72E-08	6.72E-08	6.72E-08	6.72E-08	6.72E-08	6.72E-08	6.72E-08	7.81E-08	6.72E-08	6.72E-08	1.04E-06	6.72E-08	6.69E-08
Glass	6.80E-08	6.80E-08	6.80E-08	6.80E-08	6.80E-08	6.80E-08	6.80E-08	6.80E-08	6.80E-08	6.80E-08	6.80E-08	6.80E-08	6.80E-08	3.85E-02
Wine	6.80E-08	6.80E-08	6.80E-08	6.80E-08	6.80E-08	6.80E-08	6.80E-08	6.80E-08	8.35E-03	6.80E-08	6.80E-08	6.80E-08	6.80E-08	3.88E-08
Cancer	6.80E-08	6.80E-08	6.80E-08	6.80E-08	6.80E-08	6.80E-08	6.80E-08	6.80E-08	6.80E-08	6.80E-08	7.90E-08	2.73E-01	6.80E-08	6.16E-08
CMC	6.80E-08	6.80E-08	6.80E-08	6.80E-08	6.80E-08	6.80E-08	6.80E-08	6.80E-08	6.80E-08	6.80E-08	2.22E-07	6.80E-08	6.80E-08	5.71E-08
Seeds	6.80E-08	6.80E-08	6.80E-08	6.80E-08	6.80E-08	6.80E-08	6.80E-08	6.80E-08	6.80E-08	6.80E-08	6.76E-08	6.80E-08	6.80E-08	6.39E-08
Heart	6.80E-08	6.80E-08	6.80E-08	6.80E-08	6.80E-08	6.80E-08	6.80E-08	6.80E-08	6.80E-08	6.80E-08	1.10E-05	6.80E-08	6.80E-08	4.37E-08
Survival	8.01E-09	8.01E-09	8.01E-09	8.01E-09	8.01E-09	8.01E-09	7.76E-09	8.01E-09	7.98E-09	8.01E-09	5.72E-01	8.01E-09	8.01E-09	7.02E-09

Bold indicates *p*-values greater than 0.05.

### 5.5 Visual Analysis of Clustering Results

After the above experiments, Tables 3, 4, and Figures 5, and 6 demonstrate that the QLSMFO algorithm is characterized by high accuracy, rapid convergence, and reliable stability of performance. For the purpose of showing the clustering capability of the proposed algorithm more vividly, this section will show the process of clustering in a graphical way.

#### 5.5.1 Clustering Process of QLSMFO

**FIGURE 7:**
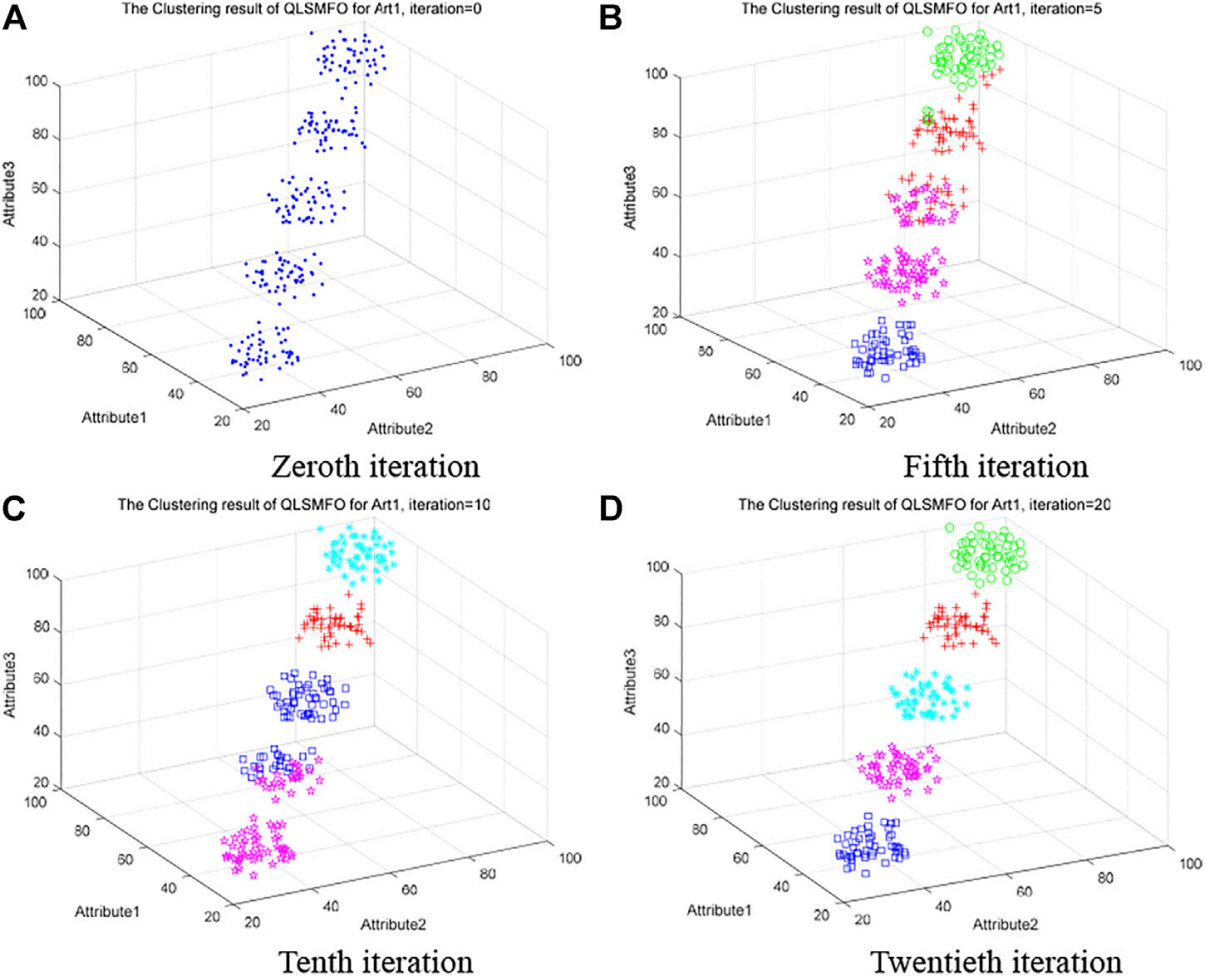
Clustering process of QLSMFO on Art 1 dataset at iteration is 0, 5, 10, and 20. **(A)** Zeroth iteration. **(B)** Fifth iteration. **(C)** Tenth iteration. **(D)** Twentieth iteration.

Artificial Dataset I will be applied to visualize the process of clustering in QLSMFO during this section. Artificial Dataset I has three attributes and five clusters. The *x*, *y*, and *z* axes correspond to three attribute values, respectively. Different types of clusters are marked by green, blue, red, magenta, and cyan. The clustering situation of QLSMFO on Artificial Dataset 1 when the number of iterations is 0, 5, 10, and 20 are presented in Figure 7. The initial distribution of the Art I dataset is presented in Figure 7A. When the number of iterations is 5, as shown in Figure 7B, there is confusion in the data classification between the green and red clusters. During the classification process, there is a misassignment of a class to the class represented by magenta and red, such as the part where magenta and red are mixed. Only blue is classified correctly. As the iterations continue, Figure 7C shows the clustering results with an iteration number of 10. Cyan, red, blue, and magenta are classified correctly, but the part of the data that mixes blue and magenta is classified incorrectly. Figure 7D demonstrates the clustering effect at 20 iterations. It is obvious from the figure that five classes are correctly classified, and there is no data confusion between classes.

The four graphs in Figure 7 vividly show the clustering process of QLSMFO on Artificial Dataset 1, and the correct classification effect is achieved in the 20th generation, which illustrates the effectiveness of the algorithm and the fast classification speed.

#### 5.5.2 Comparison of the Clustering Process with Other Algorithms

The comparison results of QLSMFO and other algorithms in Artificial Dataset 1, Artificial Dataset 2, Iris dataset, and CMC dataset will be presented in this section.

**FIGURE 8:**
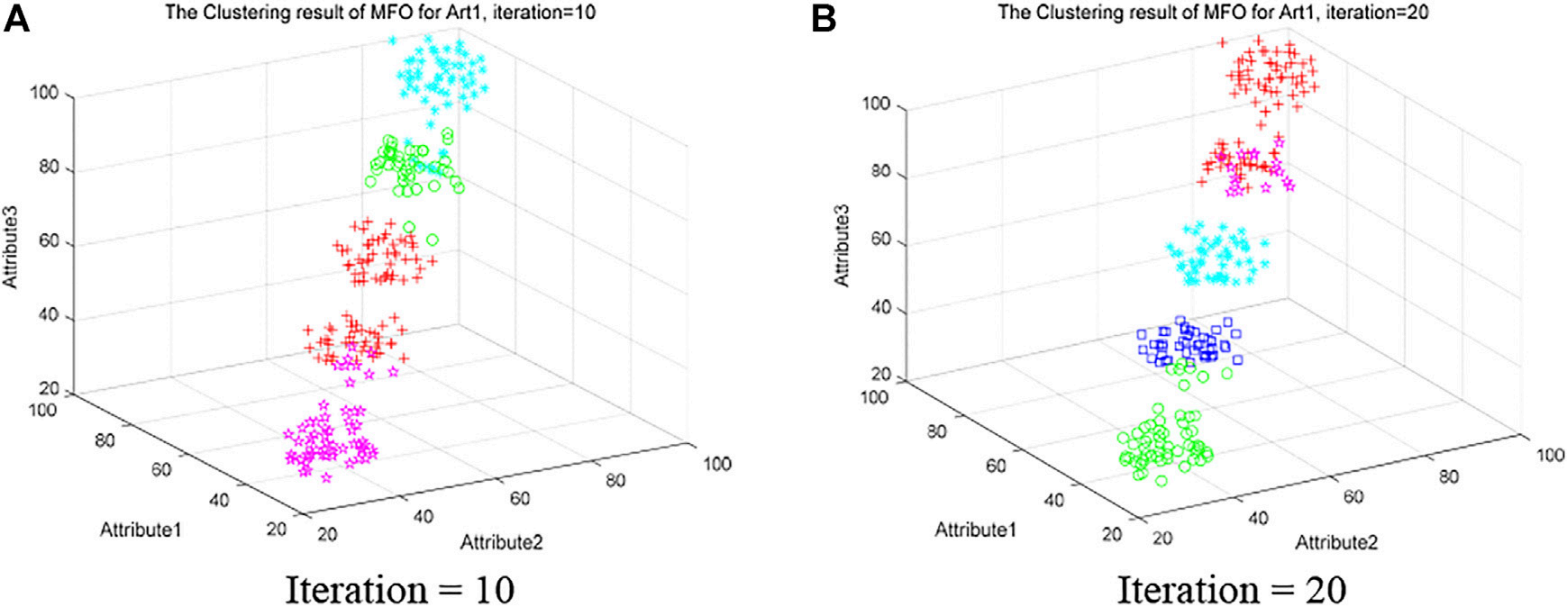
Clustering results of MFO on the Art 1 dataset at iteration is 10 and 20. **(A)** Iteration = 10. **(B)** Iteration = 20.

Figure 8 shows the clustering results of MFO on Artificial Dataset 1 with iterations of 10 and 20, respectively. In Figure 8A, the data is not successfully divided into five categories, and there is data confusion within each category cluster. Although Figure 8B divides the classes into five classes. However, most of the data in magenta are misclassified to red, and there is a small amount of data misclassification between blue and green. Compared with Figures 7C and D in Section 5.5.1, It is observed that QLSMFO has high efficiency and high precision in solving clustering problems compared to the original MFO.

To make a comparison of the results of QLSMFO with other algorithms on Artificial Dataset II, the original MFO and an algorithm ranked second to QLSMFO are selected for further comparison.

**FIGURE 9:**
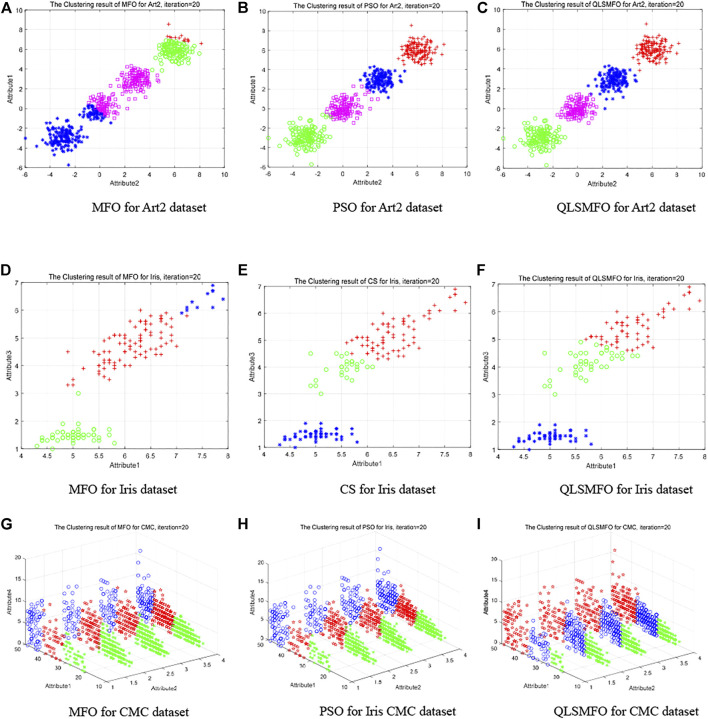
Clustering results of QLSMFO and other algorithms on different datasets at iteration 20. **(A)** MFO for Art 2 dataset. **(B)** PSO for Art 2 dataset. **(C)** QLSMFO for Art 2 dataset. **(D)** MFO for Iris dataset. **(E)** CS for Iris dataset. **(F)** QLSMFO for Iris dataset. **(G)** MFO for CMC dataset. **(H)** PSO for Iris CMC dataset. **(I)** QLSMFO for CMC dataset.

Table 3 illustrates that PSO has reached the same level as QLSMFO on Best, Worst, and Mean. PSO algorithm is selected for further performance comparison with QLSMFO since the Std. of PSO is second only to QLSMFO. The maximum iteration value is given as 20. Figures 9A–C exhibit the clustering outcomes of the 20th generation on Artificial Dataset II for MFO, PSO, and QLSMFO, respectively. It is seen from Figure 9A that the blue, magenta, green, and red in the MFO are classified incorrectly. In Figure 9B, the classification result of the PSO is better than that of the MFO, and the data has been successfully divided into four categories, but there are still some data confusions between magenta and blue that have not been successfully separated. From Figure 9C, QLSMFO is observed, the classification effect is the best, there is no data confusion between clusters, and the classification is correct. It can be clearly shown that the classification effect of QLSMFO is better.

The data in Table 3 shows that the values of CS on Best, Worst, Mean, and Std. on the Iris dataset are closest to QLSMFO. In order to further show the performance difference between QLSMFO and CS in the clustering effect, the clustering results with 20 iterations are selected for comparison. Figures 9D–F show the classification results of MFO, CS, and QLSMFO on the 20th generation of attribute 1 and attribute 3 of the Iris dataset. In Figure 9D, the red and blue data appear chaotic. In Figure 9E the green and red appear chaotic. In Figure 9F, green and red achieve a good balance, and the data are correctly separated.

Figures 9G–I indicate the clustering effect of the 20th generation of MFO, PSO, and QLSMFO on attributes 1, 2, and 4 of the CMC dataset. It is observed that the blue, red, and green borders in Figure 9I are clear and there is no mixing. In Figures 9G and H, there is a situation where the intermediate data and the data on both sides are stuck, so the effect of QLSMFO is better.

### 5.6 Verify the Effectiveness of the Three Strategies in QLSMFO

**FIGURE 10:**
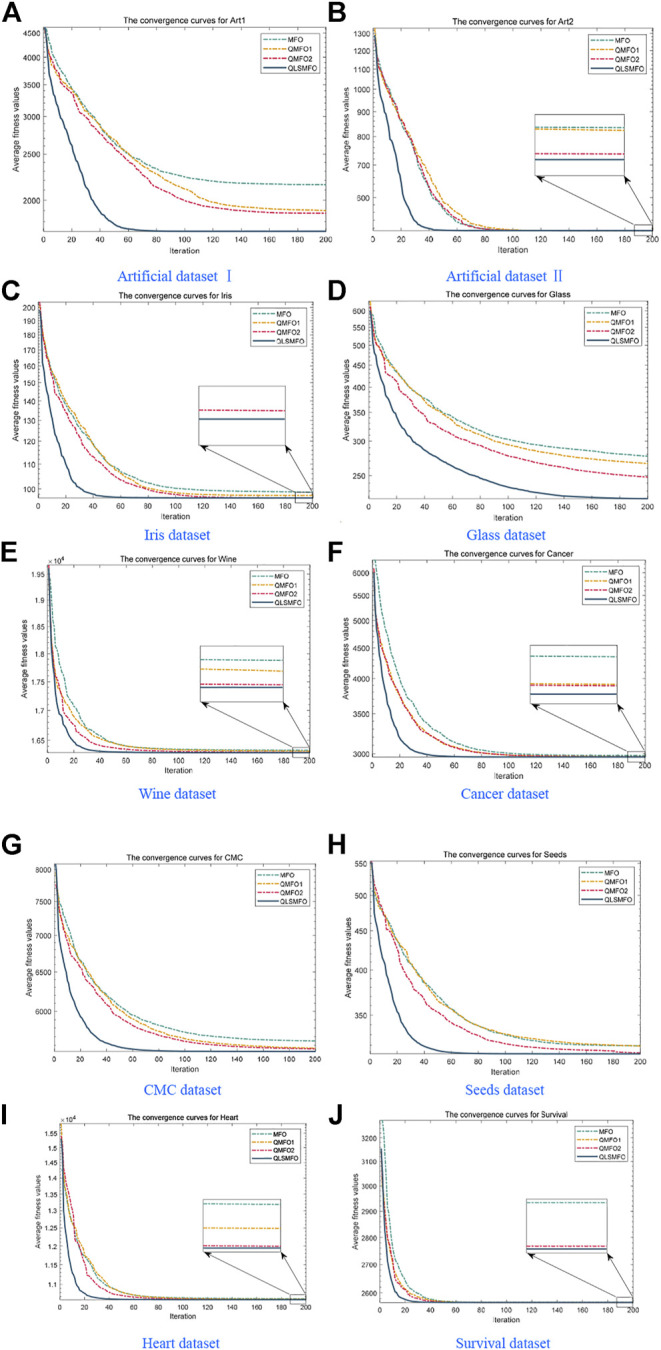
Convergence curves of MFO, QMFO1, QMFO2, and QLSMFO on the 10 datasets. **(A)** Artificial Dataset I **(B)** Artificial Dataset II. **(C)** Iris dataset. **(D)** Glass dataset. **(E)** Wine dataset. **(F)** Cancer dataset. **(G)** CMC dataset. **(H)** Seeds dataset. **(I)** Heart dataset. **(J)** Survival dataset.

In order to verify the effectiveness of the three improved strategies added to the QLSMFO algorithm, the proposed QLSMFO is compared with the original MFO, QMFO1, and QMFO2. Firstly, QMFO1 represents an improved algorithm with quantum initialization and QRG strategy added to the original MFO. Secondly, the improved algorithm formed by adding the Levy flight strategy based on the QMFO1 algorithm is named QMFO2. Finally, the algorithm that combines the three strategies is the QLSMFO. The four algorithms were tested on 10 UCI clustering datasets. The algorithm parameters and simulation experiments are consistent with the previous content. In addition, the bold in the table indicates the optimal value. The experimental data in Table 5 compares the mean and standard deviation of the original MFO, QMFO1, QMFO2, and QLSMFO to measure the performance improvement of the algorithm. The effect of the added strategies on the convergence speed of the algorithm is observed in Figure 10.

**TABLE 5:** Numerical results of improved algorithms with different strategies on 10 clustered data.

Datasets	MFO	Std.	QMFO1	Std.	QMFO2	Std.	QLSMFO	Std.
	Mean		Mean		Mean		Mean	
Art I	2.1549E+03	3.5595E+02	1.9008E+03	2.9446E+02	1.8755E+03	2.7818E+02	1.7183E+03	5.4772E-06
Art II	5.1390E+02	1.9986E-06	5.1390E+02	5.5624E-06	5.1390E+02	4.6172E-07	5.1390E+02	1.1664E-13
Iris	9.8798E+01	4.3183E+00	9.7550E+01	3.1493E+00	9.6658E+01	8.9730E-03	9.6655E+01	1.6264E-08
Glass	2.7702E+02	1.6129E+01	2.6647E+02	1.2987E+01	2.4773E+02	1.2168E+01	2.2098E+02	9.2425E+00
Wine	1.6332E+04	5.3271E+01	1.6317E+04	3.3197E+01	1.6297E+04	2.3583E+00	1.6293E+04	7.8681E-01
Cancer	2.9819E+03	2.6386E+01	2.9690E+03	1.1290E+01	2.9683E+03	1.0190E+01	2.9644E+03	2.9443E-07
CMC	5.6509E+03	1.1345E+02	5.5692E+03	4.1229E+01	5.5612E+03	3.4173E+01	5.5322E+03	9.5136E-04
Seeds	3.1926E+02	1.3420E+01	3.1911E+02	8.6249E+00	3.1269E+02	1.5788E+00	3.1180E+02	9.8831E-07
Heart	1.0648E+04	2.4114E+01	1.0634E+04	1.3657E+01	1.0624E+04	8.2164E-01	1.0623E+04	2.1012E-04
Survival	2.5684E+03	6.1684E+00	2.5670E+03	1.6420E-10	2.5671E+03	2.5730E-01	2.5670E+03	5.8086E-13

The experimental results in Table 5 show that in terms of algorithm accuracy and stability, QMFO1 has a certain improvement over the original MFO algorithm on 10 datasets, and achieves the same accuracy as QLSMFO on Art II and survival datasets. After adding quantum initialization and QRG in the original MFO algorithm, due to the increase in the diversity of the moth population, the search range is expanded and the exploration ability of the algorithm is enhanced. The QMFO1 algorithm achieves a better average value than the original MFO on all ten datasets. Since QRG can adaptively guide the algorithm to search in a more desired search direction, the stability of the algorithm is improved. It can be seen from Table 5 that the accuracy and stability of QMFO2 have been further improved on the basis of QMFO1. When implementing the Levy flight strategy, the moth population needs to be assessed to identify the poor moths and update their positions. Levy flight improves the searchability of the moth. From the convergence curves in Figures 10A–J, it can be seen that the QMFO2 algorithm has significantly improved the convergence speed. Both Table 5 and Figure 10 show that the local search strategy has the greatest contribution to improving the accuracy, stability, and convergence speed of QLSMFO, the average value, and standard deviation are both optimal, and the convergence speed is also the fastest. This is due to the superior exploitation capability of the local search strategy based on SFLA. By observing the experimental results of the four algorithms, the multi-strategy effectively improves the performance of the algorithm due to a single strategy. The quantum initialization and Levy flight strategy, both improved the performance of the original algorithm to a certain extent, and the local search strategy has greatly contributed to the accuracy, stability, and convergence speed of the algorithm.

## 6 Conclusion and Future Works

A quantum-inspired moth-flame optimizer with an enhanced local search strategy (QLSMFO) is introduced to address clustering analysis in this research. Quantum coding is used for the coding of individuals in the moth population to enrich the population diversity and thus boost the exploration capacity of the algorithm. The addition of the quantum revolving gate guides the population to evolve towards a better solution, bringing the two phases of exploration and exploitation into a state of balance. The local search strategy based on SFLA enhances the mining capability of standard MFO. Finally, the Levy flight is used to update the poor solutions in the population. This mechanism improves the population quality and accelerates the rate of convergence. To confirm the effectiveness and practical performance of QLSMFO in clustering analysis, we compared it with eleven algorithms including ABC, ALO, CS, DE, FPA, GWO, MFO, MVO, PSO, WOA, SFLA, QBA, GQPSO, and K-means on two artificial datasets and eight famous UCI datasets. Experimental results show that QLSMFO significantly outperforms comparison algorithms with regard to the accuracy, convergence speed, and robustness. Future research will try to use QLSMFO to solve higher latitude cluster analysis problems. Try to expand the application scope of QLSMFO.

## Data Availability

The original contributions presented in the study are included in the article, further inquiries can be directed to the corresponding authors.
